# Whole-Exome Sequencing Identifies a Novel *TRPM4* Mutation in a Chinese Family with Atrioventricular Block

**DOI:** 10.1155/2021/9247541

**Published:** 2021-04-17

**Authors:** Yi Dong, Ran Du, Liang-liang Fan, Jie-yuan Jin, Hao Huang, Ya-qin Chen, Dan-dong Bi, Rong Xiang

**Affiliations:** ^1^Department of Patient Service Center, Xiangya Hospital of Central South University, Changsha 410008, China; ^2^Department of Cell Biology, School of Life Sciences, Central South University, Changsha 410013, China; ^3^Department of Cardiology, The Second Xiangya Hospital of Central South University, Changsha 410078, China

## Abstract

Atrioventricular block (AVB) is a leading cause of sudden cardiac death, and most of AVB cases are presented as autosomal dominant. The electrocardiogram of AVB patients presents an abnormal progressive cardiac conduction disorder between atria and ventricles. *Transient receptor potential melastatin 4* (*TRPM4*) is a nonselective Ca^2+^-activated cation channel gene defined as a novel disease-causing gene of AVB. So far, 47 mutations of *TRPM4* have been recorded in Human Gene Mutation Database. The aim of this study was to explore the relationship between *TRPM4* mutation and pathogenesis of AVB. We investigated a Chinese family with AVB by whole-exome sequencing. An arrhythmia-related gene filtering strategy was used to analyze the disease-causing mutations. Three different bioinformatics programs were used to predict the effects of the mutation result. A novel mutation of *TRPM4* was identified (c.2455C>T/p.R819C) and cosegregated in the affected family members. The three bioinformatics programs predicted that the novel mutation may lead to damage. Our study will contribute to expand the spectrum of *TRPM4* mutations and supply accurate genetic testing information for further research and the clinical therapy of AVB.

## 1. Introduction

Inherited progressive cardiac conduction disease (PCCD) is defined as an abnormal progressive cardiac conduction disorder [1], in which atrioventricular block (AVB) is characteristic by the presence of heart conduction block between atria and ventricle on 12-lead electrocardiogram (ECG). This abnormality of cardiac conduction will elevate the risk of sudden cardiac death, which will become a heavy burden for both family and society [2–4]. AVB can be classified into three tiers according to the extension degree and ECG. First-degree AVB often shows a 0.2-second delay of the PR interval in ECG [5]. Second-degree AVB remains poorly understood as it can present in all ages without obvious symptoms. Previous studies recorded complex, variable rhythm patterns like various atrioventricular conduction and changing atrial rhythms in second-degree AVB patients [6, 7]. Varying clinical figures like tiredness, chest pain, shortness of breath, or syncope are associated with third-degree AVB patients. Besides, third-degree AVB patients generally necessitate pacemakers for survival [8, 9].

The *transient receptor potential melastatin 4* (*TRPM4*) encodes a nonselective Ca^2+^-activated cation channel which is highly expressed in Purkinje fibers and nodal tissue [10]. Activated TRPM4 channels act on membrane repolarization and impact Ca^2+^ driving force which are essential for regulating Ca^2+^ oscillation frequency in cardiomyocytes [11, 12]. Mutations in *TRPM4* were found to be associated with various inherited cardiac conduction disease such as progressive familial heart block type I (PFHB1), Brugada syndrome (BrS), right bundle branch block (RBBB), bradycardia, and AVB [13–15].

In this study, we investigated a typical phenotypic family with a history of syncope and sudden cardiac death. By applying whole-exome sequencing (WES) and arrhythmia-related gene filtering, we identified a novel heterozygous mutation (c.2455C>T/p.R819C) in *TPRM4* (NM_001195227) that might be the nosogenesis in this AVB family.

## 2. Materials and Methods

### 2.1. Subjects

This study was approved by the review board of Xiangya Hospital of the Central South University. The proband and the relatives who participated in the study have been given informed consent letters. Blood was collected from the proband and related family members. The proband was examined by standard 12-lead ECGs.

### 2.2. Whole-Exome Sequencing

Genomic DNA was extracted with a DNeasy blood and tissue kit (Qiagen, Valencia, CA). Exome capture, high-throughput sequencing, and common filtering were delivered to the Novogene Bioinformatics Institute (Beijing, China). All the exomes were captured by means of Agilent SureSelect Human All Exon V6 kits and were sequenced by an Illumina HiSeq2000 platform. Filtering strategies were the same as our previous study [16, 17].

### 2.3. Mutation Analysis

PolyPhen-2, SIFT, and MutationTaster bioinformatics programs were used to predict the effects of the mutations detected by WES. The mutation which was most likely to lead to the disease was verified by Sanger sequencing. Segregation analysis was performed among all the family members. Primer pairs were designed by the PrimerQuest Tool (http://sg.idtdna.com/Primerquest/Home/Index), and the sequences of primers will be provided upon request.

## 3. Results

### 3.1. Clinical Subjects

**Figure 1:**
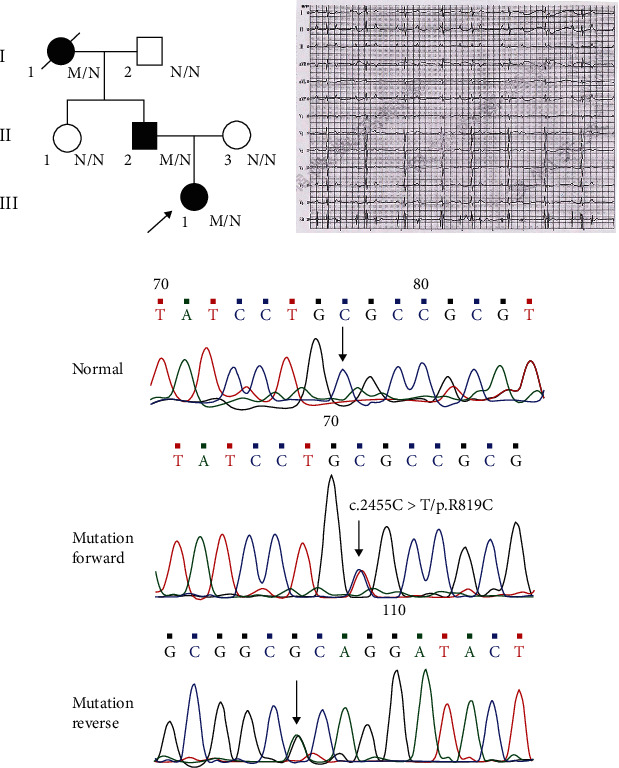
Description of the family with AVB. (a) Pedigree of the family. Family members are identified by generations and numbers. Squares = males; circles = females; black symbols = affected individuals; white symbols = unaffected individuals; arrow = the proband; M/N = people carried *TRPM4* mutation; N/N = people without *TRPM4* mutation. (b) The 12-lead ECGs of the proband. (c) Sanger sequencing results of the TRPM4 mutation. Sequence chromatogram indicates a C to T transition of nucleotide 2455.

We identified a Chinese family with AVB (Figure 1(a)). The proband (III-1) is a 10-year-old girl from Hunan province, which presented dizziness for 4 days. She was sent to Xiangya Hospital due to syncope. The 12-lead ECGs showed a high-grade atrioventricular conduction block (Figure 1(b)). She was diagnosed as third-degree AVB. Besides, other two family members (I-1 and II-2) also had a history of AVB. Moreover, her grandmother (I-1) died of sudden cardiac death (Table 1).

### 3.2. Genetic Analysis

To identify the potential gene mutation leading the AVB in this family, WES was applied. WES yielded 10.92 Gb data with 99.7% coverage of target region, and 98.7% of targets were covered over 10x. After alignment and single-nucleotide variant (SNV) calling, 54725 variants were found in this family. Data filtering excluded shared common variants from the 1000 Genomes Project, YH, dbSNP132, and ESP databases; 487 unique SNPs were identified. Arrhythmia-related genes were used to filter candidate mutations in which 10 mutations were identified (Table S1). All filtered variants were predicted by 3 different bioinformatics programs (Table 2). Based on the prediction results, among all of the 10 mutations, only *TRPM4* (c.2455C>T/p.R819C) was regarded as the disease-causing gene by all the bioinformatics programs. The value of MAF of the novel mutation (p.R819C) in the Asian population was zero. Therefore, we infer that the *TRPM4* mutation is the pathogenic cause of the proband.

**Figure 2:**
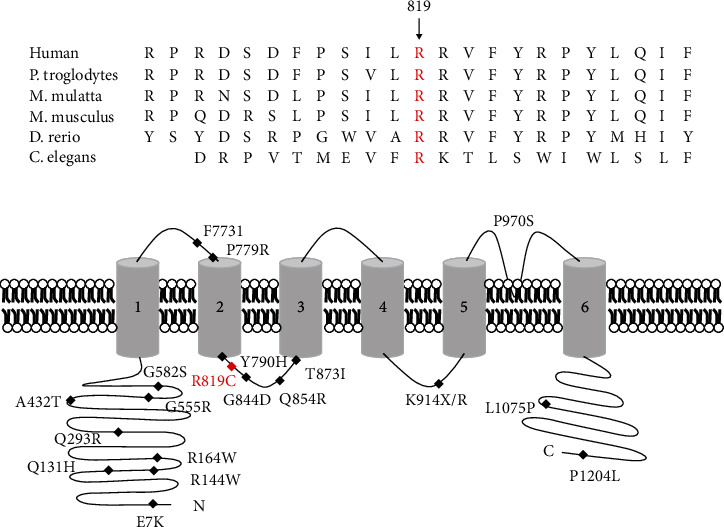
The bioinformatics analysis and summary of *TRPM4* mutations. (a) Alignment of multiple TRPM4 protein sequences across species. The R819 affected amino acid locates in the highly conserved amino acid region in different mammals (from Ensembl). Red words show the R819 site. (b) Schematic diagram of TRPM4, all the domains, and location of known mutations are labeled. The novel mutation is shown in red. The mutations identified in AVB were shown in blue.

To further confirm the *TRPM4* mutation is responsible for the AVB, Sanger sequencing was employed to examine the mutation segregated within this family. The result indicated that the novel missense mutation of *TRPM4* is cosegregated with the affected AVB members in this family, but not with the normal members (Figure 1(c)). Alignment analysis of TRPM4 amino acid sequences from human, mouse, rat, etc., showed that the site was highly conserved (Figure 2(a)). It is a further indication that the *TRPM4* (c.2455C>T/p.R819C) variant lead to AVB among this family.

## 4. Discussion

In this study, a proband with a high-grade atrioventricular conduction block and a history of syncope was explored by WES combined with arrhythmia-related gene filtering. A novel missense mutation *TRPM4* (c.2455C>T/p.R819C) was identified, which is located in the exon 18. This missense mutation was further confirmed in other family members by Sanger sequencing, which was accounted for the cosegregation of the members with the disease phenotype.

TRPM4 plays a crucial role in the cardiac conduction system. Immunohistochemistry results show that TRPM4 is highly enriched in ventricular cardiomyocytes and is highest in Purkinje fibers [18]. In previous studies, Mathar et al. have provided that TRPM4 knock-out mice resulted in a shortened ventricular action potential and an increased *β*-adrenergic-dependent ventricular systole [19]. In 2015, Jacobs et al. revealed the silence of TRPM4 mice appeared severe cardiac muscle necrosis by obstructing the left anterior descending artery [20]. The current study further supported the idea of the role of TRPM4 in AVB. A developed ventricular fibrillation and idioventricular rhythm had been recorded in overexpression of TRPM4 [21]. Thus, our study regards TRPM4 mutation as the result of AVB among 10 identified mutations.

In 2009, Kruse et al. revealed the first TRPM4 mutation in progressive familial heart block type I patient. E7K, a gain of function variant, would cause an elevated TRPM4 channel density and affect the sensibility of Small Ubiquitin MOdifier conjugation (SUMOylation) [22]. Up till now, 47 mutations in *TRPM4* have been recorded in the Human Gene Mutation Database (http://www.hgmd.cf.ac.uk/ac/index.php). Most of these mutations are located in the cytoplasmic N-terminus domain, which is important for Ca^2+^ sensitivity, desensitization, and voltage dependence of the TRPM4 channel (Figure 2(b)) [18]. According to previous studies, mutations (Y790R and G844D) mapped to the first intracellular loop showed a gain of function in TRPM4. Besides, cotransfection experiment of G844D with Ubc9 displayed an increased current density which will lead to abnormal SUMOylation and dysfunction of endocytosis [23, 24]. Our novel mutation (c.2455C>T/p.R819C) of *TRPM4* is located in a highly conserved domain, which shares a common intracellular loop with G844D. Thus, we suppose that the alternation from alkaline amino acid (Arg) to polar but uncharged amino acid (Cys) may also affect the channel density at cell surface like G844D in TRPM4. The increased cell surface conductance may affect electrical signal transmission along the Purkinje fibers. The abnormal action potential propagation causes cardiac conduction disorder [25]. Currently, mutations in TRPM4 were identified in autosomal recessive Brugada syndrome and Progressive Symmetric Erythrokeratodermia (PSEK). As Wang et al. discussed, most of the mutations that caused PSEK were located in the S6 transmembrane segment of TRPM4. This may keep an effective channel for electrical signal transmission [10, 26].

## 5. Conclusions

In conclusion, we identify a novel TRPM4 mutation (c.2455C>T/p.R819C) in a two-generation family with AVB by genetic sequencing. The present study, in which a novel mutation of *TRPM4* was identified, not only further explain the possible cause of AVB in the family but also expand the spectrum of *TRPM4* mutations and contribute to genetic diagnosis and counseling for families with AVB.

## Figures and Tables

**Table 1:** Demographic and clinic features in members of the family.

Family members	Sex	Age (year)	EGC	History	Mutation
Proband (III-1)	Female	10	High-grade AVB	Syncope	c.2455C>T
II-1	Female	35	—	—	—
II-2	Male	40	PR interval delayed	Syncope	c.2455C>T
II-3	Female	37	—	—	—
I-1	Female	34^∗^	—	Sudden death	—
I-2	Male	66	—	—	—

^∗^Age of death.

**Table 2:** Variants identified by WES in combination with cardiomyopathy-related gene filter in the family.

CHR	POS	RB	AB	Gene name	AA change	MutationTaster	PolyPhen-2	SIFT	ACMG statement
chr1	100342136	G	A	AGL	AGL:NM_000645:exon9:c.G1355A:p.R452Q	Disease causing (1)	Probably damaging (0.902)	Tolerated (0.065)	BS4
chr1	228527780	C	T	OBSCN	OBSCN:NM_001098623:exon70:c.C17393T:p.T5798M	Polymorphism (0.977)	Probably damaging (0.877)	Damaging (0.024)	BS4
chr6	170598799	G	A	DLL1	DLL1:NM_005618:exon2:c.C152T:p.P51L	Polymorphism (0.998)	Benign (0.037)	Tolerated (0.71)	BP4
chr7	154379618	G	C	DPP6	DPP6:NM_001290253:exon6:c.G886C:p.V296L	Disease causing (1)	Benign (0.033)	Damaging (0)	BS4
chr11	2906376	A	C	CDKN1C	CDKN1C:NM_000076:exon1:c.T344G:p.V115G	Polymorphism (1)	Benign (0.011)	Tolerated (0.089)	BP4
chr12	116445394	T	C	MED13L	MED13L:NM_015335:exon11:c.A2060G:p.Q687R	Polymorphism (0.902)	Benign (0.001)	Tolerated (0.859)	BP4
chr19	49703979	C	T	TRPM4	TRPM4:NM_001195227:exon18:c.C2455T:p.R819C	Disease causing (1)	Damaging (1)	Damaging (0)	PM2, PP1, PP3, PM1
chr20	10393636	G	A	MKKS	MKKS:NM_018848:exon3:c.C527T:p.A176V	Polymorphism (1)	Benign (0.011)	Tolerated (0.256)	BP4
chr20	10393860	G	T	MKKS	MKKS:NM_018848:exon3:c.C303A:p.N101K	Polymorphism (0.965)	Benign (0.324)	Tolerated (0.216)	BP4
chr21	43531589	G	C	UMODL1	UMODL1:NM_001199527:exon11:c.G2041C:p.D681H	Polymorphism (1)	Benign (0.007)	Damaging (0.011)	BP4

CHR = chromosome; POS = position; RB = reference sequence base; AB = alternative base identified.

## Data Availability

The data supporting the conclusions are included in the article. The datasets used and/or analyzed during the current study are available from the corresponding author upon reasonable request.

## Supplementary Materials

Supplementary Materials Table S1: the arrhythmia-related gene list. All the genes in the list have been reported to involve in the occurrence of arrhythmia. It was used to filter the proband's candidate mutations.
