# Supplementary material for: Whole-Exome Sequencing Identifies a Novel TRPM4 Mutation in a Chinese Family with Atrioventricular Block
Source: Biomed Res Int. 2021 Apr 17;2021:9247541. doi: 10.1155/2021/9247541 (PMC8075657; doi:10.1155/2021/9247541)
Supplement: Supplementary Materials — Table S1: the arrhythmia-related gene list. All the genes in the list have been reported to involve in the occurrence of arrhythmia. It was used to filter the proband's candidate mutations. [file 9247541.f1.docx]

Table S1. The arrhythmia-related gene list.

| GPD1L | KCNJ8 | MYL2 | NKX2-5 | POLR2M | SLC25A4 | TMEM43 | XIRP1 |
| --- | --- | --- | --- | --- | --- | --- | --- |
| HCN4 | KCNQ1 | MYL3 | NODAL | PPP3B | SLC2A10 | TMOD1 | XIRP2 |
| HDAC1 | KRAS | MYL5 | NOS1AP | PPP3CA | SLC8A1 | TMPO | ZIC3 |
| HDAC2 | LAMA4 | MYL7 | NOTCH1 | PPP3R1 | SLMAP | TNNC1 |  |
| HRAS | LAMP2 | MYLK | NPPA | PRKAG2 | SMAD3 | TNNI3 |  |
| ILK | LBD3 | MYLK2 | NRAP | PRKCE | SMYD1 | TNNT1 |  |
| ITGB1BP2 | LEFTY2 | MYLK3 | NRAS | PTPN11 | SMYD2 | TNNT2 |  |
| JAG1 | LIMS1 | MYO6 | NRG1 | RAF1 | SNTA1 | TPM1 |  |
| JPH2 | LIMS2 | MYOM1 | OBSCN | RANGRF | SOS1 | TPM2 |  |
| JUP | LMCD1 | MYOM2 | OBSL1 | RBM20 | SYNE1 | TPM3 |  |
| KBTBD13 | LMNA | MYOT | PAK1 | RYR1 | SYNE2 | TRDN |  |
| KCNA5 | MAP2K1 | MYOZ1 | PALLD | RYR2 | SYNM | TRIM54 |  |
| KCND3 | MAP2K2 | MYOZ2 | PARVB | SCN1B | TAZ | TRIM55 |  |
| KCNE1 | MIB1 | MYOZ3 | PDE5A | SCN2B | TBX20 | TRIM63 |  |
| KCNE2 | MIR208 | MYPN | PDLIM1 | SCN3B | TBX5 | TRPM4 |  |
| KCNE3 | MYBPC3 | MYZAP | PDLIM3 | SCN4B | TCAP | TTN |  |
| KCNE5 | MYH11 | NEB | PDLIM5 | SCN5A | TGFB2 | TTR |  |
| KCNH2 | MYH6 | NEBL | PDLIM7 | SDHA | TGFB3 | TXNRD2 |  |
| KCNJ2 | MYH7 | NEURL2 | PKP2 | SGCD | TGFBR1 | UNC45B |  |
| KCNJ5 | MYH7B | NEXN | PLN | SHOC2 | TGFBR2 | VCL |  |
